# Prognostic Value of Tumor Heterogeneity on 18F-FDG PET/CT in HR+HER2− Metastatic Breast Cancer Patients receiving 500 mg Fulvestrant: a retrospective study

**DOI:** 10.1038/s41598-018-32745-z

**Published:** 2018-09-27

**Authors:** Yannan Zhao, Cheng Liu, Yingjian Zhang, Chengcheng Gong, Yi Li, Yizhao Xie, Bingrui Wu, Zhongyi Yang, Biyun Wang

**Affiliations:** 1 Department of Medical Oncology, Fudan University Shanghai Cancer Center, Department of Oncology, Shanghai Medical College, Fudan University, Shanghai, China 200032; 2 Department of Nuclear Medicine, Fudan University Shanghai Cancer Center, Department of Oncology, Shanghai Medical College, Fudan University, Center for Biomedical Imaging, Fudan University, Shanghai Engineering Research Center of Molecular Imaging Probes, Shanghai, China 200032; 3 Key Laboratory of Nuclear Physics and Ion-beam Application (MOE), Fudan University, Shanghai, China 200433; 4 Key Laboratory of Glycoconjugate Research Ministry of Public Health, Department of Biochemistry and Molecular Biology, Shanghai Medical College, Fudan University, Shanghai, China

## Abstract

Heterogeneity has been demonstrated to be a predictor of treatment failure and drug resistance. Our study aimed to investigate imaging parameters, including tumor heterogeneity, as prognostic factors of response to 500 mg fulvestrant using 18F-FDG PET/CT. Twenty-seven estrogen receptor (HR)-positive/HER2-negative metastatic breast cancer patients who received 500 mg fulvestrant and underwent 18F-FDG PET/CT before treatment were retrospectively included. In PET/CT scans, conventional parameters (maximum and mean standardized uptake value, metabolic tumor volume [MTV], total lesion glycolysis [TLG]) and heterogeneity parameters (intra-tumor heterogeneity index [HI] and inter-tumor heterogeneity coefficient of variation [COV]) were analyzed. Progression-free survival (PFS) was mainly assessed for efficacy. The survival analyses were performed using the Kaplan–Meier method. Univariate and multivariate analysis were performed using the Cox proportional hazard model. Univariate analysis indicated that a high SUVmax and a high tumor HI at baseline were associated with longer PFS of fulvestrant (P = 0.036 and P = 0.033, respectively). Liver metastasis, SUVmax and HI were statistically significant in multivariate analysis (P values of 0.017, 0.025 and 0.043, respectively). 18F-FDG based intra-tumor heterogeneity appears to be a potential predicator of efficacy of fulvestrant among HR+HER2− metastatic breast cancer patients.

## Introduction

Breast cancer is the most common malignancy in Chinese women^1^. 20–30% patients finally experience advanced or metastatic breast cancer though receiving standard adjuvant therapy^2^. Luminal subtype (hormone receptor [HR]+/HER2−) formed a large proportion of metastatic breast cancer^3^, and endocrine therapy reveals the cornerstone of the treatment in these patients^4^. However, not all patients will response to endocrine therapy and predictive factors are needed to distinguish patients who will benefit or not.

Metastatic breast tumors often show high intra-tumor and inter-tumor heterogeneity in genetic condition, biological and metabolic behaviors. Heterogeneity is therefore a prognostic factor^5^ and may be a predictor of treatment failure and drug resistance, including endocrine resistance. Recently, 18F-FDG PET/CT is used to evaluate tumor heterogeneity by tumor textural information and glucose uptake^6–8^. It is an easy and non-invasive method to assess heterogeneity compared with genomic approach by biopsy. Previous studies show that imaging classifiers are associated with event-free survival (EFS), prognostic outcome and drug resistance^6,9,10^. These classifiers include maximum standard uptake value (SUVmax), mean SUV (SUVmean), metabolic volume measurements (MTV), total lesion glycolysis (TLG), which reflect the level of FDG uptake. Overall, high FDG uptake is associated with poor prognostic factors such as worse survival^11,12^ as well as endocrine resistance^10^.

Previous studies focused on association between efficacy of endocrine therapy and the parameters of FDG uptake^10^, but not tumor heterogeneity. Tumor heterogeneity can be measured as an 18F-FDG PET/CT parameter and evaluated as heterogeneity index (HI) and coefficient of variation (COV). This study is aimed to assess imaging parameters of 18F-FDG based tumor heterogeneity as prognostic factor of response to endocrine therapy using 18F-FDG PET/CT. We here explore these imaging biomarkers and patient characteristics as prognostic factor of progression-free survival (PFS) for 500 mg fulvestrant in HR+/HER2− breast cancer patients.

## Materials and Methods

### Patients

Study population consisted of 27 consecutive patients with HR+HER2− advanced breast cancer who were treated with 500 mg fulvestrant in Fudan University Shanghai Cancer Center and underwent fluorodeoxyglucose (FDG) PET/CT within 4 weeks before treatment between Jan 2011 and Dec 2015. All data were retrospectively collected from the medical records. Advanced breast cancer is defined as unresectable, locally advanced breast cancer, de novo stage IV breast cancer, and recurrent MBC. ER+/PR+ was defined as tumor tissue that expressed estrogen or progesterone receptors in ≥10% of the cells, according to the local laboratory parameters. HER2- was defined as immunohistochemistry (IHC) 0–1+ or immunohistochemistry (IHC) 2+ and fluorescence *in situ* hybridisation (FISH)−, where appropriate. Tumor tissue included primary breast cancer or metastatic tumor tissue. Patients with HER2 over-expression or gene amplification were excluded from this study. Patients with only bone metastasis were excluded as heterogeneity seems to be extreme in bone lesions to influence the analysis.

This study was approved by the Fudan University Shanghai Cancer Center Ethic Committee and Institutional Review Boards for clinical investigation. All of the methods were performed in accordance with the Declaration of Helsinki and the relevant guidelines. All of the patients signed written informed consent forms before study.

### PET/CT scans

18F-FDG was automatically generated by using Explora FDG4 module on a cyclotron (Siemens CTI RDS Eclipse ST, Knoxville, Tennessee, USA). The purity of radiochemical was over 95%. All patients were required to fast for at least 6 h. Blood glucose level of each patient was measured before the administration of 18F-FDG (dosage: 7.4 MBq/kg), and it should not exceed 10 mmol/L at the time of injection. The patients were comfortably lay down in a quiet and dimly lit room before and after the injection. All patients were scanned on the same instrument. All PET/CT image scans were performed on a Siemens biograph 16HR PET/CT in a 3-dimension, high resolution mode (the transaxial intrinsic spatial resolution was 4.1 mm, full-width at half-maximum in the center of the field of view) after 60 mins after injection. The process of data acquisition was as follows: For attenuation correction, CT scanning was first acquired using a low-dose technique [120 kV, CARE Dose (Siemens), 80–250 mA, pitch 3.6, rotation time 0.5] from the proximal thighs to head. A PET emission scan covering the same transverse field of view was obtained immediately after the CT scan (2–3 minutes/bed). We used a gaussian-filter iterative reconstruction method (iterations 4; subsets 8; image size 168) for the reconstruction of emission images.

### Image analysis

Two board certified nuclear medicine physicians with over 5-year experiences analyzed the images independently on a multimodality computer platform (Syngo, Siemens, Knoxville, Tennessee, USA). They reached a consensus if there were inconsistent or equivocal interpretations.

Semiquantitative analysis of tumor glucose metabolic activity was obtained by using SUV based on body weight. The maximum and mean SUV (SUVmax, SUVmean) for each lesion were recorded by manually placing an individual ROI around each tumor on all consecutive slices that contained the lesion on coregistered and fused transaxial PET/CT images. Besides, MTV was also recorded. It was automatically extracted from the manual delineation using software based on an adaptive threshold method. The threshold was defined as SUV ≥ 2.5. The voxels presenting SUV intensity >2.5 within the contouring margin were included to define the MTV. The TLG was obtained by using the following formula: TLG = SUVmean × MTV. A quantitative measure of intratumoral heterogeneity, heterogeneity index (HI) was acquired by dividing SUVmax by SUVmean for metastatic disease^13,14^. Inter-tumoral heterogeneity was evaluated as coefficient of variation (COV). The COV of metastatic lesions was calculated from SUVmax of every ROIs as the ratio of the standard deviation to the mean^9^.

### Treatment and follow-up

Fulvestrant was administered by intramuscular injection in a 500 mg regimen that incorporates a day 14 loading element (500 mg on days 0, 14, and 28, and every 28 days thereafter). All the premenopausal patients received concurrent luteinising hormone-releasing hormone analogues (LHRHa). The patients received treatment until disease progression, intolerable toxicity, or voluntary refusal.

Clinical follow-up and response assessment included conventional imaging (e.g. CT, MRI, bone scan), serum tumor markers, and evaluation of symptoms, as deemed appropriate by the treating physician. Tumor assessment was performed every three month until disease progression or death occurred and was identified according to RECIST 1.1. The efficacy outcomes included progression free survival (PFS), objective response rate (ORR), clinical benefit rate (CBR) and overall survival (OS). PFS was defined as the time from fulvestrant treatment to objective disease progression or death for any cause before documented disease progression. ORR was defined as proportion of patients with complete or partial response. CBR was defined as proportion of all patients with complete response, partial response, or stable disease for at least 24 weeks. OS was defined as time interval from fulvestrant treatment to death in follow-up.

### Statistical analysis

All distributions were expressed as median for quantitative data or count (percentage) for categorical data. We selected the median as the cut-off value for imaging parameters. Kaplan-Meier plots revealed median PFS with corresponding 95% confidence intervals and P values for all patients. Associations between baseline tumor PET-derived images parameters (SUVmax, SUVmean, MTV, TLG, HI, and COV) values/demographic factors and PFS were examined with the log-rank test. Univariate analysis was estimated using COX proportional hazards model and expressed as hazard ratio with corresponding 95% confidence intervals and P values. A multivariate Cox proportional hazard model was developed using stepwise regression (forward selection) to explore independent prognostic factor of PFS. Effects of variables were expressed as hazard ratios with corresponding 95% confidence intervals and P values. All image parameters and significant demographic factors in the univariate analysis entered into the model. The enter limit and remove limit were P = 0.10 and P = 0.15, respectively. Univariate analysis for CBR were conducted using logistic regression analysis. Evaluated lesions included all metastatic lesions with SUV ≥ 2.5 but excluded bone lesions. All analyses were two-sided and P values below or equal to 0.05 were considered statistically significant. Data was analyzed by SPSS 19.0 software (IBM Corporation, Armonk, NY, USA).

## Result

### Patient characteristics and treatment outcome

The demographics and clinical characteristics of the patients were showed in Table 1. In our study, the median follow-up period was 15.6 months (range: 0.73–59.3). The number of tumors identified by FDG PET was 2 to 12, with a median of 4 tumors per patient. At cutoff date of the data collection (Aug, 2017), 16 patients experienced progression and 5 patients had died among 27 patients. Median PFS was 9.4 months (95%CI 4.0–14.8) and median OS was not reached. ORR was 7.4%(2/27) and CBR was 55.6% (15/27).

**Table 1:** Patient characteristics.

Characteristics	No. (%)
Median age, years(range)	59 (37–78)
Advanced or metastatic	
De novo stage IV	2 (7.4)
Metastatic	25 (92.6)
DFI	
≤24 mo	4 (14.8)
>24 mo	21 (77.8)
No. of metastatic sites	
1	3 (11.1)
2	10 (37.0)
≥3	14 (51.9)
Metastatic sites	
Visceral	15 (55.6)
Liver	6 (22.2)
Lung	12 (44.4)
Non-visceral	12 (44.4)
Prior palliative chemotherapy	
Yes	8 (29.6)
No	19 (70.4)
Lines of endocrine therapy	
1	19 (70.4)
2	4 (14.8)
≥3	4 (14.8)

### Relation between baseline tumor characteristics and PFS

Among 27 metastatic breast cancer patients, most patients (14/27) had more than 2 metastatic sites and 6 patients (22.2%) had liver metastases. 19 patients (70.3%) received 500 mg fulvestrant as first-line endocrine therapy for MBC. 8 patients (28.6%) had been treated with palliative chemotherapy for MBC.

**Figure 1:**
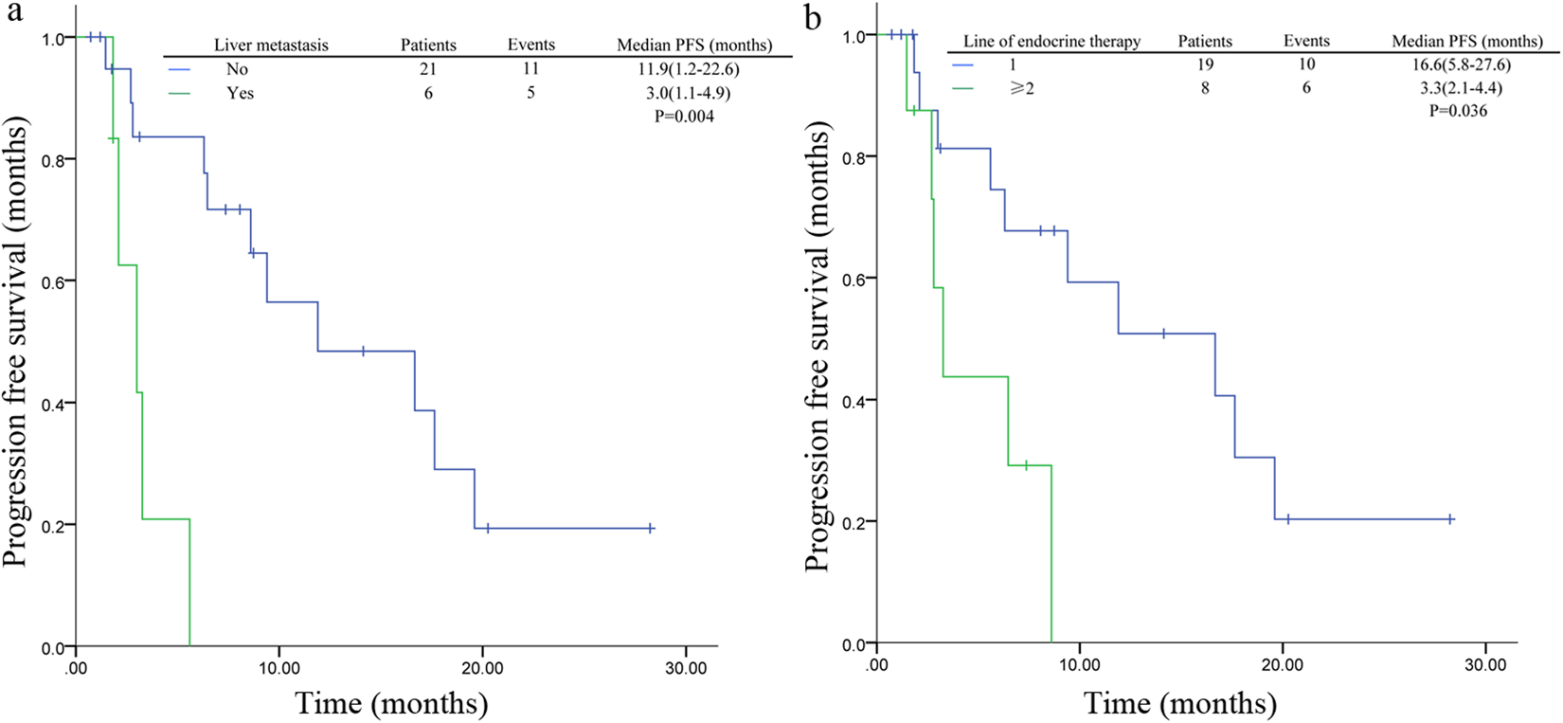
Kaplan–Meier curves for progression-free survival in patients with different tumor characteristics. (**a**) For patients stratified by liver metastasis. (**b**) For patients stratified by line of endocrine therapy. Abbreviations: CI, confidence interval; PFS, progression-free survival.

Patients with no liver metastasis had a longer PFS compared to those with liver metastasis (HR = 8.9, 95%CI 2.1–38.7, P = 0.004, Fig. 1a). Median PFS was 3.0 months vs 11.9 months in patients with or without liver metastasis. Patients who received prior endocrine therapy for MBC demonstrated a worse PFS compared to those who did not (Fig. 1b). Median PFS was 16.9 months in patients with first-line endocrine therapy, while 3.3 months in patients with subsequent endocrine therapy (HR = 3.8, 95%CI 1.1–12.9, P = 0.029). Number of metastatic sites and prior palliative chemotherapy were not significantly related to PFS (Table 2).

**Table 2:** Summary of progression-free survival analysis (bone only metastasis patients excluded).

Parameters	No.	Event	Median survival	Log-rank	Univariate analysis		Multivariate analysis	
			(95%CI)	P value	HR(95%CI)	P value	HR(95%CI)	P value
No. of metastatic sites								
1	3	2	19.6 (Not reached)	0.65	1.1 (0.6–2.2)	0.66		
2	10	7	6.3 (0.0–14.0)					
≥3	14	7	6.5 (0.0–13.8)					
Liver metastasis								
Yes	6	5	3.0 (1.1–4.9)	0.001	8.9 (2.1–38.7)	0.004*	5.6 (1.1–27.0)	0.017*
No	21	11	11.9 (1.2–22.6)					
Line of endocrine therapy for MBC								
1	19	10	16.6 (5.8–27.6)	0.019	3.8 (1.1–12.9)	0.029*		
≥2	8	6	3.3 (2.1–4.4)					
Prior palliative chemotherapy								
Yes	8	4	6.5 (0–15.7)	0.95	0.97 (0.3–3.0)	0.95		
No	19	12	9.4 (3.1–15.7)					
COV								
<0.275	14	8	8.6 (0.3–16.9)	0.55	0.7 (0.3–2.0)	0.55		
≥0.275	13	8	9.4 (2.4–16.4)					
HI								
<2.05	13	9	5.6 (0.45–10.7)	0.027	0.26 (0.07–0.9)	0.036*	0.69 (0.13–0.94)	0.043*
≥2.05	14	7	16.7 (8.6–24.7)					
SUVmax								
<6.09	14	10	5.6 (1.3–9.9)	0.026	0.32 (0.1–0.91)	0.033*	0.50 (0.15–0.84)	0.025*
≥6.09	13	6	17.3 (11.4–23.9)					
SUVmean								
<3.92	13	9	5.6 (1.7–9.4)	0.189	0.51 (0.18–1.4)	0.19		
≥3.92	14	7	16.7 (7.2–26.1)					
MTV(ml)								
<18.78	13	8	6.3 (0–14.5)	0.438	0.67 (0.24–1.8)	0.44		
≥18.78	14	8	16.7 (3.8–29.6)					
TLG(g)								
<72.5	13	8	6.3 (0–13.6)	0.419	0.66 (0.24–1.8)	0.42		
≥72.5	14	8	16.7 (3.8–29.6)					

Abbreviations: CI, confidence interval; PFS, progression-free survival. COV, coefficient of variation. HI, heterogeneity index.

SUVmax, maximum standard uptake value. SUVmean, mean SUV. MTV, metabolic volume measurements. TLG, total lesion glycolysis. *P<0.05.

### Relation between PET-derived parameters and PFS

**Figure 2:**
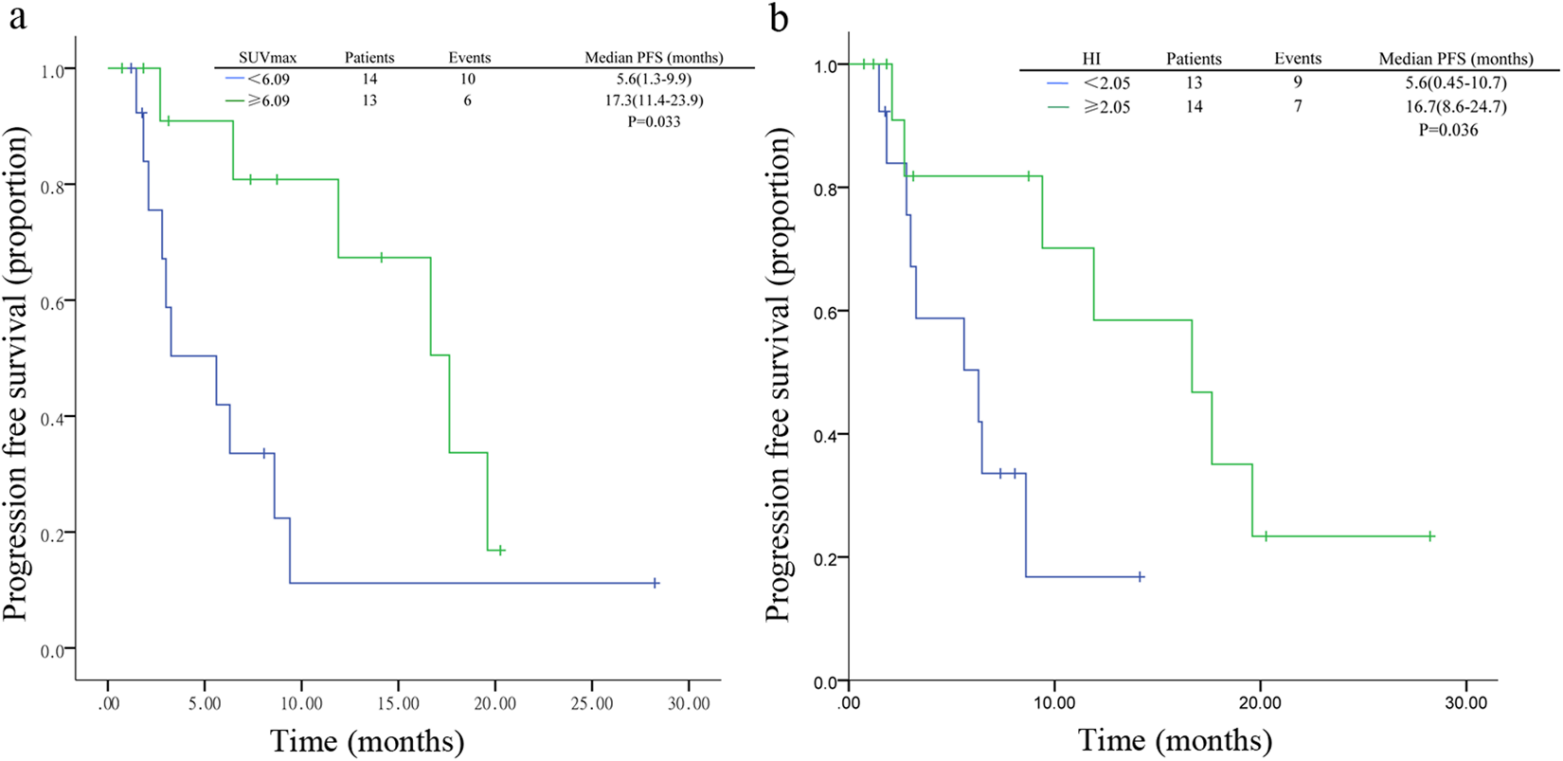
Kaplan–Meier curves for progression-free survival in patients with different imaging parameters. (**a**) For patients stratified by SUVmax. (**b**) For patients stratified by HI. Abbreviations: CI, confidence interval; PFS, progression-free survival. SUVmax, maximum standard uptake value. HI, heterogeneity index.

The cut-off value of SUVmax, SUVmean, MTV, HI, COV and TLG determined by median value of each value were 6.1, 3.9, 18.8 ml, 2.0, 0.27 and 72.5 g, respectively. Most demographic factors were balanced in different groups of imaging groups except non-visceral metastasis in MTV (Table S1). Univariate analysis indicated high SUVmax at baseline was associated with longer PFS (HR = 0.32, 95%CI 0.10–0.91, P = 0.033, Fig. 2a). The median PFS was 17.3 months in patients with baseline tumor SUVmax ≥ 6.1 and 5.6 months in those with SUVmax < 6.1. In addition, patients with high tumor HI revealed a significantly longer PFS compared with those with low HI (HR = 0.26, 95%CI 0.074–0.91, P = 0.036, Fig. 2b). The median PFS were 16.7 and 5.6 months in patients with HI < 2.05 and HI ≥ 2.05, respectively. Multivariate analysis showed that liver metastasis, SUVmax and HI were independent predictive value of PFS (P = 0.017, 0.043 and 0.025, respectively, Table 2).

However, SUVmean, tumor volume parameters and COV were not predictive for PFS, with no significant P value was observed by log-rank methods (P = 0.19 for SUVmean; P = 0.44 for MTV, P = 0.419 for TLG, P = 0.55 for COV; Table 2). We also adjusted for the non-visceral metastasis in multivariate analysis and found MTV was still insignificant (data not shown).

### Relation between PET-derived parameters and CBR

Patients with high SUVmax achieved a higher CBR compared with those with low SUVmax (OR = 6.0, 95%CI 1.1–32.5, P = 0.038, Table S2). CBR was 76.9% in patients with baseline tumor SUVmax ≥ 6.1 and 35.7% in those with SUVmax < 6.1.

## Discussion

Heterogeneity is a key feature of malignant tumor and is an important mechanism for drug resistance. 18F-FDG PET/CT proved to be a novel method to assess tumor heterogeneity and heterogeneity parameters of PET/CT are predictive markers for recurrence, survival and chemo-resistance. It is unclear if the heterogeneity parameters are associated with the effectiveness of endocrine therapy. Our study was designed to evaluate the prognostic value of novel PET parameters based on tumor heterogeneity in HR+HER2− metastatic breast cancer patients who were treated with endocrine therapy. In this study, we retrospectively analyzed 27 HR+HER2− MBC patients who received 500 mg fulvestrant and underwent 18F-FDG PET/CT within 4 weeks before treatment.

In our study, patients with high baseline 18F-FDG tumor uptake had longer PFS at cut-off value of 6.09 (P = 0.033). The median PFS was 17.3 months in patients with baseline tumor SUVmax ≥ 6.1 (vs 5.6 months in those with SUVmax < 6.1). HI was associated with PFS with a cut-off value of 2.05 (P = 0.036). Patients with high HI revealed longer PFS, compared with those with low HI. Furthermore, other tumor characteristics, like liver metastases and lines of endocrine therapy were significant prognostic factor of PFS.

However, our study reported a controversial result compared with previous studies. Groheux *et al*. found that high SUV, including SUVmax, SUVmean and SUVpeak, were associated with shorter EFS^8^. However, homogeneity was not predictive for EFS (HR = 2.27, P = 0.07). Kurland *et al*. explored imaging biomarkers of PFS in breast cancer patients receiving endocrine therapy^10^. Endocrine therapy mainly included tamoxifen, aromatase inhibitor and fulvestrant. FDG combined with FES PET/CT predicted the efficacy of endocrine therapy, showing that patients with low FDG uptake had a longer median PFS, patients with high FDG uptake and high average FES uptake had a moderate median PFS and patients with high FDG uptake and low average FES uptake had a shorter median PFS (26.1, 7.9 months and 3.3 months, respectively). Overall, high FDG uptake predicts a worse prognosis and efficacy for endocrine therapy.

We suppose the possible reasons as follows: 1) 17β-estradiol (E2) increased glucose uptake capacity in an HR-positive breast cancer cell line mediated by ER-dependent activation of PI3K/Akt signaling pathway^15,16^. High glucose metabolism may reflect improved activation of ER signaling pathway, which is an important proliferation pathway in ER+ breast cancer. Therefore, fulvestrant, as a selective estrogen receptor downregulator, may reveal an enhanced inhibition to ER signaling pathway in patients with high FDG uptake. 2) Nearly one third patients (8/27) received prior endocrine therapy and chemotherapy before fulvestrant treatment and baseline 18F-FDG PET/CT scan, which may influence the FDG uptake, SUV value and heterogeneity. Among patients with prior treatments, metastatic lesions, which were responsive to previous treatments, may reduce glucose uptake and vascularization, leading to less blood perfusion and hypoxia. As a result, lesions with low FDG uptake may have decreased perfusion and receive less fulvestrant compared with the lesions with sufficient perfusion. In addition, hypoxia may enhance the resistance to drugs. Therefore, patients with high SUV value may respond well to fulvestant and have a longer PFS when receiving fulvestrant.

Previous studies show imaging heterogeneity markers of PET have a better predictive power on therapy response and outcome in different cancers, such as rectal cancer, non–small cell lung cancer and nasopharyngeal carcinoma^6,7,9^. Most of them focused on patients who were naïve to systemic treatments after diagnosis of primary tumor or metastatic disease. We first explored the importance of heterogeneity parameters by 18F-FDG PET/CT among patients with metastatic breast cancer and prior treatments. We evaluated the intra-tumor and inter-tumor heterogeneity by HI and COV, respectively. We found that only intra-tumor heterogeneity was significantly associated with PFS. It is generally believed that one metastatic lesion grows from a cell to a colony dependent on clonal expansion. Intra-tumor heterogeneity may develop during this process. It may better reflect the metabolism status and response to therapies, especially in pretreated lesions. Alterations resulting in resistance may occur during treatment^17,18^. Despite the elimination of responsive clones, the resistant clones may reduce treatment success and lead to therapy failure^19^.

We conducted all patients’ analysis including those with bone metastasis (Tables S3 and 4. Patients with only bone metastasis were excluded as heterogeneity seems to be extreme in bone lesions to influence the analysis. Bone metastasis includes osteolytic and osteosclerotic bone metastasis and PET/CT is more sensitive to osteolytic bone metastasis^20^. Prior treatment may also increase18F-FDG PET/CT based tumor heterogeneity^21^. Heterogeneity of biological behavior of bone metastasis and detection bias may influence the accuracy of imaging parameters. Therefore, these patients and lesions were excluded in our study.

Some limitations should be considered in our study. First, our study has a limited size population, which makes it difficult to detect a significant statistical difference. We retrospectively analyzed the patients with fulvestrant treatment and baseline 18F-FDG PET/CT scan and only a small number of patients met the criteria and were included in this study. A larger number of patients are needed to further identify the significance of heterogeneity parameters. Second, patient characteristics are heterogeneous in terms of prior treatments, metastatic sites and DFI (disease free survival) and the bias are unavoidable. More homogeneous patients and prospective study are needed. Third, since we conducted the study among HR+HER2− metastatic breast cancer patients, evaluating heterogeneity of tumor lesions by FES PET may better reveal activity of HR signaling pathway. Previous study showed that 18F-FES uptake has a significant positive correlation with the ER expression and can predict tumor response to endocrine therapy in ER+ breast cancer in an ER+ xenograft model, but not for 18F-FDG. Little has been reported about the prediction of FDG PET-CT and FES PET-CT for endocrine therapy efficacy in breast cancer patients. Yet, Kurland *et al*. demonstrated that patients with low FDG uptake had the longer PFS compared with those with high FDG uptake. And FES uptake predicted PFS but only in patients with high FDG uptake. It reported that the PFS of endocrine therapy were 26.1, 7.9 and 3.3 months, respectively in ER+ breast cancer patients with low FDG uptake, high FDG and FES uptake and high FDG but low FES uptake. These all indicated that 18F-FDG joining with 18F-FES imaging may better predict the efficacy of endocrine therapy and further studies validating their prediction power are ongoing by our center and other researchers (NCT02398773).

## Conclusion

This study was a pioneer work, providing preliminary data of 18F-FDG based intra-tumor heterogeneity appears to be a potential predicator of efficacy of fulvestrant among HR+HER2− metastatic breast cancer patients. These findings provide new methods to help the endocrine treatment of patients with HR+HER2− metastatic breast cancer.

## Electronic supplementary material

Supplementary Dataset 4

## Data Availability

The datasets generated during and/or analyzed during the current study are available from the corresponding author on reasonable request.
