# Supplementary material for: Prognostic Value of Tumor Heterogeneity on 18F-FDG PET/CT in HR+HER2− Metastatic Breast Cancer Patients receiving 500 mg Fulvestrant: a retrospective study
Source: Sci Rep. 2018 Sep 27;8:14458. doi: 10.1038/s41598-018-32745-z (PMC6160449; doi:10.1038/s41598-018-32745-z)
Supplement: Supplementary file 1 — Supplementary Dataset 4 [file 41598_2018_32745_MOESM1_ESM.docx]

**Prognostic Value of Tumor Heterogeneity on 18F-FDG PET/CT in HR+HER2- Metastatic Breast Cancer Patients receiving 500mg Fulvestrant: a retrospective study**

Yannan Zhao^1^#, Cheng Liu^2, 3^#, Yingjian Zhang^2, 3^, Chengcheng Gong^1^,Yi Li^1^, Yizhao Xie^1^, Bingrui Wu^4^, Zhongyi Yang^2, 3^, Biyun Wang^1^

^1^Department of Medical Oncology, Fudan University Shanghai Cancer Center, Department of Oncology, Shanghai Medical College, Fudan University, Shanghai, China 200032

^2^ Department of Nuclear Medicine, Fudan University Shanghai Cancer Center, Department of Oncology, Shanghai Medical College, Fudan University, Center for Biomedical Imaging, Fudan University, Shanghai Engineering Research Center of Molecular Imaging Probes, Shanghai, China 200032

^3^Key Laboratory of Nuclear Physics and Ion-beam Application (MOE), Fudan University, Shanghai, China 200433

^4^Key Laboratory of Glycoconjugate Research Ministry of Public Health, Department of Biochemistry and Molecular Biology, Shanghai Medical College, Fudan University, Shanghai, China.

Correspondence to: E-mail: yangzhongyi21@163.com (ZY)

wangbiyun0107@hotmail.com (BW)

# These authors contributed equally to this work.

**Supplementary Tables**

| **Table S1. Patient characteristics divided by imaging parameters (N=27)** | | | | | | | | | |
| --- | --- | --- | --- | --- | --- | --- | --- | --- | --- |
| Characteristics | COV＜0.275 | COV≥0.275 | P value | HI＜2.05 | HI≥2.05 | P value | SUVmax＜6.09 | SUVmax≥6.09 | P value |
|  | No.(%) | No.(%) |  | No.(%) | No.(%) |  | No.(%) | No.(%) |  |
| Median age, years(range) | 59.5(37-69) | 58(41-78) |  | 55(37-64) | 59(41-78) |  | 55(37-69) | 61(41-78) |  |
| Advanced or metastatic |  |  |  |  |  |  |  |  |  |
| De novo stage IV | 1 | 1 | 0.96 | 1 | 1 | 0.95 | 1 | 1 | 0.96 |
| Metastatic | 13 | 12 |  | 12 | 13 |  | 13 | 12 |  |
| DFI |  |  |  |  |  |  |  |  |  |
| ≤24 mo | 3 | 1 | 0.30 | 4 | 0 | 0.08 | 3 | 1 | 0.30 |
| ＞24 mo | 10 | 11 |  | 8 | 13 |  | 10 | 11 |  |
| No. of metastatic sites |  |  |  |  |  |  |  |  |  |
| 1 | 2 | 1 | 0.61 | 2 | 1 | 0.70 | 1 | 2 | 0.87 |
| 2 | 6 | 4 |  | 4 | 6 |  | 6 | 4 |  |
| ≥3 | 6 | 8 |  | 7 | 7 |  | 7 | 7 |  |
| Metastatic sites |  |  |  |  |  |  |  |  |  |
| Visceral |  |  |  |  |  |  |  |  |  |
| Liver | 4 | 2 | 0.42 | 4 | 2 | 0.31 | 5 | 1 | 0.09 |
| Non-visceral | 6 | 6 | 0.87 | 4 | 8 | 0.18 | 5 | 7 | 0.35 |
| Prior palliative chemotherapy |  |  |  |  |  |  |  |  |  |
| Yes | 10 | 9 | 0.90 | 9 | 10 | 0.90 | 11 | 8 | 0.34 |
| No | 4 | 4 |  | 4 | 4 |  | 3 | 5 |  |
| Lines of endocrine therapy |  |  |  |  |  |  |  |  |  |
| 1 | 10 | 9 | 0.90 | 7 | 12 | 0.08 | 10 | 9 | 0.90 |
| ≥2 | 4 | 4 |  | 6 | 2 |  | 4 | 4 |  |
| (continued) |  |  |  |  |  |  |  |  |  |
| Characteristics | SUVmean＜3.92 | SUVmean≥3.92 | P value | MTV＜18.78 | MTV≥18.78 | P value | TLG＜72.5 | TLG≥72.5 | P value |
|  | No.(%) | No.(%) |  | No.(%) | No.(%) |  | No.(%) | No.(%) |  |
| Median age, years(range) | 55(37-69) | 61(41-78) |  | 58(40-68) | 57.5(37-78) |  | 58(40-68) | 61(37-78) |  |
| Advanced or metastatic |  |  |  |  |  |  |  |  |  |
| De novo stage IV | 1 | 1 | 0.96 | 0 | 2 |  | 0 | 2 | 0.16 |
| Metastatic | 12 | 13 |  | 13 | 12 |  | 13 | 12 |  |
| DFI |  |  |  |  |  |  |  |  |  |
| ≤24 mo | 3 | 1 | 0.25 | 1 | 3 | 0.25 | 2 | 2 | 0.93 |
| ＞24 mo | 9 | 12 |  | 12 | 9 |  | 11 | 10 |  |
| No. of metastatic sites |  |  |  |  |  |  |  |  |  |
| 1 | 2 | 1 | 0.75 | 2 | 1 | 0.11 | 2 | 1 | 0.01 |
| 2 | 5 | 5 |  | 7 | 3 |  | 8 | 2 |  |
| ≥3 | 6 | 8 |  | 4 | 10 |  | 3 | 11 |  |
| Metastatic sites |  |  |  |  |  |  |  |  |  |
| Visceral |  |  |  |  |  |  |  |  |  |
| Liver | 5 | 1 | 0.06 | 3 | 3 | 0.92 | 3 | 3 | 0.92 |
| Non-visceral | 5 | 7 | 0.55 | 9 | 3 | 0.013* | 8 | 4 | 0.09 |
| Prior palliative chemotherapy |  |  |  |  |  |  |  |  |  |
| Yes | 9 | 10 | 0.90 | 10 | 9 | 0.48 | 11 | 8 | 0.12 |
| No | 4 | 4 |  | 3 | 5 |  | 2 | 6 |  |
| Lines of endocrine therapy |  |  |  |  |  |  |  |  |  |
| 1 | 8 | 11 | 0.34 | 9 | 10 | 0.90 | 9 | 10 | 0.90 |
| ≥2 | 5 | 3 |  | 4 | 4 |  | 4 | 4 |  |

| **Table S2. Summary of clinical benefit rate analysis (N=27)** | | |  |
| --- | --- | --- | --- |
| Parameters | CBR(%) | OR(95%CI) | P value |
| COV |  |  |  |
| ＜0.275 | 57.1 | 0.87(0.19-4.0) | 0.86 |
| ≥0.275 | 53.8 |  |  |
| HI |  |  |  |
| ＜2.05 | 46.1 | 2.1(0.45-9.8) | 0.86 |
| ≥2.05 | 64.2 |  |  |
| SUVmax |  |  |  |
| ＜6.09 | 35.7 | 6.0(1.1-32.5) | 0.038* |
| ≥6.09 | 76.9 |  |  |
| SUVmean |  |  |  |
| ＜3.92 | 46.1 | 2.1(0.45-9.8) | 0.34 |
| ≥3.92 | 64.2 |  |  |
| MTV(ml) |  |  |  |
| ＜18.78 | 46.1 | 2.1(0.45-9.8) | 0.34 |
| ≥18.78 | 64.3 |  |  |
| TLG(g) |  |  |  |
| ＜72.5 | 46.2 | 2.1(0.45-9.8) | 0.34 |
| ≥72.5 | 64.3 |  |  |

Abbreviations: CI, confidence interval; PFS, progression-free survival. COV, coefficient of variation. HI, heterogeneity index.

SUVmax, maximum standard uptake value. SUVmean, mean SUV. MTV, metabolic volume measurements. TLG, total lesion glycolysis.[20]

| **Table S3. Patient characteristics including bone only metastasis patients (N=41)** | |
| --- | --- |
| Characteristics | No.(%) |
| Median age, years(range) | 59(37-78) |
| Advanced or metastatic |  |
| De novo stage IV | 3(7.3) |
| Metastatic | 38(92.7) |
| DFI |  |
| ≤24 mo | 8(18.6) |
| ＞24 mo | 30(69.8) |
| No. of metastatic sites |  |
| 1 | 11(26.9) |
| 2 | 16(39.0) |
| ≥3 | 14(34.1) |
| Metastatic sites |  |
| Visceral | 15(36.6) |
| Liver | 6(14.6) |
| Non-visceral | 26(63.4) |
| Bone only | 14(34.1) |
| Prior palliative chemotherapy |  |
| Yes | 11(73.2) |
| No | 30(26.8) |
| Lines of endocrine therapy |  |
| 1 | 27(65.9) |
| 2 | 6(14.6) |
| ≥3 | 8(19.5) |
|  |  |

| **Table S4. Summary of progression-free survival analysis (bone only metastasis patients included)** | | | | | | | | |  |
| --- | --- | --- | --- | --- | --- | --- | --- | --- | --- |
|  |  |  | Median survival | | Log-rank | Univariate analysis | | Multivariate analysis | |
| Parameters | No. | Event | (95%CI) | | P value | HR(95%CI) | P value | HR(95%CI) | P value |
| Liver metastasis |  |  |  | |  |  |  |  |  |
| Yes | 6 | 5 | 3.0(1.1-4.9) | | 0.00 | 7.5 (2.2-25.3) | 0.001* | 10.3(2.0-52.0) | 0.005* |
| No | 35 | 19 | 10.9(6.9-14.8) | |  |  |  |  |  |
| Bone only metastasis |  |  |  | |  |  |  |  |  |
| Yes | 14 | 8 | 7.2 (4.1-10.2) | | 0.57 | 1.1 (0.5-2.7) | 0.75 |  |  |
| No | 27 | 16 | 9.4 (4.0-14.8) | |  |  |  |  |  |
| Site of metastasis |  |  |  |  |  |  |  |  |  |
| Visceral | 15 | 10 | 6.4(0.0-14.6) | | 0.48 | 1.3(0.6-3.0) | 0.48 |  |  |
| Non-visceral | 26 | 14 | 8.6(5.7-11.5) | |  |  |  |  |  |
| Line of endocrine therapy for MBC |  |  |  | |  |  |  |  |  |
| 1 | 27 | 14 | 11.9(4.0-19.7) | | 0.08 | 2.2 (0.9-5.3) | 0.087 |  |  |
| ≥2 | 14 | 10 | 7.2(5.0-9.4) | |  |  |  |  |  |
| Prior palliative chemotherapy |  |  |  | |  |  |  |  |  |
| Yes | 11 | 5 | 10.9(2.1-19.6) | | 0.41 | 0.66 (0.2-1.8) | 0.41 |  |  |
| No | 30 | 19 | 7.9(4.5-11.2) | |  |  |  |  |  |
| COV |  |  |  | |  |  |  |  |  |
| ＜0.255 | 20 | 11 | 8.6(0.5-16.7) | | 0.82 | 1.09 ( 0.5-2.5) | 0.82 |  |  |
| ≥0.255 | 21 | 13 | 7.9(4.4-11.5) | |  |  |  |  |  |
| HI |  |  |  | |  |  |  |  |  |
| ＜2.05 | 20 | 10 | 5.6(0.0-11.2) | | 0.45 | 0.73 (0.32-1.6) | 0.46 |  |  |
| ≥2.05 | 21 | 14 | 11.9(6.3-17.5) | |  |  |  |  |  |
| SUVmax |  |  |  | |  |  |  |  |  |
| ＜6.35 | 21 | 9 | 5.6(0.6-10.6) | | 0.16 | 0.57 (0.25-1.3) | 0.2 |  |  |
| ≥6.35 | 20 | 15 | 11.9(4.7-19.1) | |  |  |  |  |  |
| SUVmean |  |  |  | |  |  |  |  |  |
| ＜4.18 | 21 | 10 | 6.5(3.2-9.7) | | 0.21 | 0.55 (0.24-1.3) | 0.16 |  |  |
| ≥4.18 | 20 | 14 | 11.9(10.3-13.5) | |  |  |  |  |  |
| MTV(ml) |  |  |  | |  |  |  |  |  |
| ＜35.23 | 21 | 11 | 7.2(5.3-9.1) | | 0.49 | 0.75 (0.3-1.7) | 0.49 |  |  |
| ≥35.23 | 20 | 13 | 11.4(4.9-17.9) | |  |  |  |  |  |
| TLG(g) |  |  |  | |  |  |  |  |  |
| ＜127.4 | 21 | 11 | 7.9(4.9-10.9) | | 0.54 | 0.77 (0.346-1.7) | 0.54 |  |  |
| ≥127.4 | 20 | 13 | 11.4(2.4-20.4) | |  |  |  |  |  |

Abbreviations: CI, confidence interval; PFS, progression-free survival. COV, coefficient of variation. HI, heterogeneity index.

SUVmax, maximum standard uptake value. SUVmean, mean SUV. MTV, metabolic volume measurements. TLG, total lesion glycolysis.[20]
